# Challenging Evolutionary Paradigms: *Daphnia* Populations Resurrected From Unpolluted Environments Show Enhanced Detoxification Ability to Aromatic Pollutants

**DOI:** 10.1111/mec.70272

**Published:** 2026-02-17

**Authors:** Florian Gigl, Muhammad Abdullahi, Sam Benkwitz‐Bedford, Niamh Eastwood, Jiarui Zhou, Henner Hollert, Luisa Orsini

**Affiliations:** ^1^ Department of Evolutionary Ecology and Environmental Toxicology, Faculty of Biological Sciences Goethe University Frankfurt am Main Germany; ^2^ School of Biosciences and Centre for Environmental Research and Justice (CERJ) University of Birmingham Birmingham UK; ^3^ Department Environmental Media Related Ecotoxicology Fraunhofer Institute for Molecular Biology and Applied Ecology IME Schmallenberg Germany; ^4^ LOEWE Centre for Translational Biodiversity Genomics (LOEWE‐TBG) Frankfurt am Main Germany; ^5^ The Alan Turing Institute, British Library London UK

**Keywords:** aquatic, detoxification pathways, endocrine disruption, gut microbiome, metabolism, phenanthrene, transcriptome, waterflea

## Abstract

Understanding how organisms respond to chemical stress requires disentangling genetically encoded (constitutive) adaptations from environmentally induced (plastic) responses. This challenge is particularly acute for polycyclic aromatic hydrocarbons (PAHs), widespread aquatic pollutants with well‐documented toxicity, where mechanisms of tolerance, including host–microbiome interactions, are unexplored. We used *Daphnia magna* , a keystone freshwater species with clonal reproduction and dormant egg banks to test population‐specific (constitutive) responses to phenanthrene (PHE), a common PAH. Populations resurrected from contrasting historical environments were exposed to sub‐lethal PHE concentrations, and both host transcriptomes and gut microbiomes were profiled to assess induced responses. Transcriptomic analysis revealed distinct, population‐specific responses in detoxification, stress signalling, and endocrine regulation. Unexpectedly, the semi‐pristine (pollution‐naïve) population showed higher tolerance, with robust induction of cytochrome P450 and hormonal pathways, while populations historically exposed to pollution exhibited chronic stress signatures and reduced plasticity. Gut microbiome profiling revealed PHE‐induced functional shifts across populations, with the pollution‐naïve population showing broader stress‐associated responses and historically exposed populations to pollutants exhibiting more detoxification‐focused microbiome profiles. Both host and microbial datasets consistently showed enrichment in pyruvate and carbon metabolism, indicating coordinated energy mobilisation and detoxification responses. Our results show that historical exposure to chemical stress and wider pollution does not necessarily confer enhanced physiological tolerance to PHE. Instead, hydrocarbon stress elicits coordinated, functionally linked responses across the host and its associated microbiome. By leveraging *Daphnia*'s unique ecology and evolutionary history, we disentangle constitutive from plastic responses and show that microbiome functional reconfiguration under PHE exposure is coordinated with host responses, contributing to population‐specific profiles.

## 1 Introduction

Polycyclic aromatic hydrocarbons (PAHs) are persistent environmental contaminants primarily produced by the incomplete combustion of carbon‐rich materials. While they can arise from natural sources such as volcanic activity, wildfires, and the geochemical transformation of ancient organic matter, human activities constitute the dominant source of PAHs in the environment (Du and Jing 2018; Kieta et al. 2022; Kozak et al. 2017; Slezakova et al. 2013; Xie et al. 2006). PAHs accumulate in soils, sediments, and biological tissues, where they can persist for long periods of time. Their semi‐volatile nature enables atmospheric transport and deposition across multiple environmental compartments, including remote regions (Baran et al. 2017; Friedman et al. 2014; Pirsaheb et al. 2020).

Phenanthrene (PHE) is one of the most common PAHs found in the environment (Du and Jing 2018; Gad 2014; Gorshkov et al. 2021; Kozak et al. 2017) and predominant in surface and groundwater, reflecting its high mobility and solubility (Zhang et al. 2019). Due to its ubiquitous presence both in water and sediment, PHE is found in almost all water ecosystems. Its concentration in lake sediments varies globally ranging from as little as 0.159 ng/g in minimally impacted African lakes to as high as 33,090 ng/g in Chinese Lakes (Du and Jing 2018).

PAHs are toxic to a broad range of freshwater species, including diatoms, gastropods, mussels, crustaceans, and fish. Their effects range from sublethal effects at low concentrations (≈0.1–10 μg L^−1^) to acute toxicity and mortality at higher concentrations (tens to hundreds of μg L^−1^), depending on compound, species, life stage, and exposure duration (Bhagat et al. 2016; Oliveira et al. 2007; Wu et al. 2015; Zindler et al. 2016). Aquatic organisms exposed to PAHs can experience effects ranging from developmental abnormalities and reduced growth to impaired reproduction and increased mortality (Honda and Suzuki 2020; Incardona et al. 2004; Meador et al. 2006; Raymond et al. 2021).

Chronic exposure to PAHs in fish can lead to impaired reproductive success, genotoxicity, developmental deformities, immune suppression, and histopathological damage to liver and gill tissues (Carls et al. 2003, 2008; Ramachandran et al. 2006; Van der Oost et al. 2003). Immunotoxic and endocrine‐disrupting effects have also been observed (Arukwe 2001; Reynaud and Deschaux 2006). Biomolecular impacts of PAHs in fish include the dysregulation of 7‐ethoxyresorufin O‐deethylase activity and cytochrome P450 enzymes, which often result in lipid peroxidation (LPO), the formation of reactive oxygen species (ROS) and subsequent oxidative stress (Brinkmann et al. 2013; Carls et al. 2003; Ravi et al. 2022; Shimada and Fujii‐Kuriyama 2004; Van der Oost et al. 2003; Whyte et al. 2000). Invertebrates exposed to PAHs show delayed development and oxidative stress responses. These physiological effects have been linked to the upregulation of detoxification enzymes such as cytochrome P450 monooxygenases and glutathione *S*‐transferases, indicative of cellular damage (Gan et al. 2021; Ravi et al. 2022; Turja et al. 2020). In these species, PAHs can also interfere with hormonal signalling and gametogenesis, reducing reproductive success (Banni et al. 2009). In addition, neurotoxicity has been reported in some invertebrate species, with alterations in acetylcholinesterase activity affecting neuromuscular coordination and behaviour (Xu et al. 2024).

In zooplankton, such as copepods and waterfleas, PAH exposure has been shown to reduce fecundity, impair mobility, delay development, and increase mortality (Gigl et al. 2024; Wernersson 2004). These physiological impacts can be accompanied by the upregulation of detoxification enzymes, such as cytochrome P450 monooxygenases and glutathione S‐transferases (GSTs), leading to higher oxidative stress and associated cellular damage (Zhou et al. 2020). In phytoplankton, PAH exposure can impair photosynthetic efficiency, disrupting energy metabolism (Kamalanathan et al. 2021; Othman et al. 2018).

PAHs also have known toxicological effects on humans, who are primarily exposed via inhalation, ingestion of contaminated food and drinking water, and dermal absorption (Boström et al. 2002; Kim et al. 2013), with several PAHs classified as carcinogens (Jameson 2021; Luch 2005).

The impact of PAHs extends to microbial communities in both aquatic and terrestrial ecosystems, as well as to gut microbial communities. Exposure to PAHs can reduce diversity, alter community composition, and impair metabolic functions of free‐living microbes (Castillo‐Ilabaca et al. 2024; Lin et al. 2023; Picariello et al. 2020). PAHs have also been shown to have adverse effects on the gut microbiota composition of different species. In fish, reduction of gut microbial diversity caused by PAH exposure leads to higher infection susceptibility (Colin et al. 2022; Giatsis et al. 2015); in invertebrates, PAH exposure impacts the gut microbiota by reducing the immune response and the ability to degrade toxic compounds (Adamovsky et al. 2018; Jaffar et al. 2022; Li et al. 2021). While the general toxicity of PAHs is well established, their biomolecular effects on freshwater invertebrates remain poorly understood. Sub‐lethal impacts at the cellular and molecular levels are particularly underexplored. As these species are central to aquatic food webs, PAH exposure can lead to cascading effects through bioaccumulation and biomagnification. In addition, growing evidence suggests that host‐associated microbiota play an important role in modulating stress responses, metabolism, and immunity, highlighting complex interactions between pollutants and host–microbe systems (Michaudel and Sokol 2020; Rea et al. 2016). This interplay calls for in depth analysis not only of host response to PAHs but also of their associated microbiota.

The waterflea *Daphnia*, widely used as sentinel species in ecotoxicology and central to freshwater aquatic food webs, offers a valuable model for addressing this knowledge gap. Their transparent bodies, short generation times, and well‐characterised transcriptome and microbiome (Chaturvedi et al. 2023) make them ideal for studying both host and gut microbial responses to chemical stressors (Abdullahi, Li, et al. 2022; Abdullahi, Zhou, et al. 2022; Asselman et al. 2017; Cuenca Cambronero et al. 2018; Lampert 2006; Suppa et al. 2020). Capitalising on these properties, we examined the sub‐lethal effects of PHE—the most common PAH in freshwater—on *Daphnia* and its gut microbiota to assess ecological risks and identify potential biomarkers of toxicity. A key advantage of using *Daphnia* is its ability to remain dormant for long periods of time, enabling access to historical populations with different pollution exposure histories (Cuenca Cambronero et al. 2018). This is important because previous studies have shown that populations with a history of chemical exposure often exhibit reduced tolerance to new pollutants, with potential cascading effects across the food web (Abdullahi, Zhou, et al. 2022; Gigl et al. 2024; Rogalski 2015).

In a previous study, we performed acute and chronic toxicity assays using PHE on *Daphnia* populations separated in time and with different histories of exposure to pollution (Gigl et al. 2024). Acute toxicity tests revealed marked differences in sensitivity among strains and populations, with the pollutant‐naïve population showing the highest physiological tolerance to PHE, as reflected by higher effect concentrations (EC_10_, EC_25_ and EC_50_) values relative to populations historically exposed to pollution. Chronic exposure at the population‐median EC_10_ (428 μg L^−1^) identified in acute assays resulted in significant developmental abnormalities, reduced growth, and delayed reproduction, with these fitness‐linked endpoints showing significantly greater costs in historically exposed populations than in the pollutant‐naïve population. These differences suggested broad physiological disruption and potential dysregulation at the molecular level. Notably, the pollutant‐naïve population experienced smaller fitness costs across multiple life‐history traits than populations with a history of chemical exposure, challenging the long‐standing assumption that prior chemical exposure generally confers greater tolerance to chemical stress.

Building on our previous work, this study investigates the molecular mechanisms underlying physiological responses to chemical stress in the same *Daphnia* populations with differing histories of environmental exposure. Specifically, transcriptomic and gut microbiome analyses were conducted on individuals sampled during the chronic exposure experiment (sub‐lethal PHE exposure: 428 μg/L) to identify variation in gene expression and microbial functional potential underpinning life‐history response, including development, growth, and reproduction (Gigl et al. 2024). Our goal was to identify both host and microbial pathways associated with PHE exposure and discover how these can be affected by historical exposure to pollution. The results provide biomarkers for PHE, advance our understanding of the ecological consequences of PAH contamination in freshwater ecosystems, and reveal how historical stress exposure may shape chemical tolerance in aquatic species.

## 2 Materials and Methods

### 2.1 Daphnia Culturing and Exposure

In this study, we used *Daphnia magna* populations with well‐characterised histories of environmental stress exposure. These populations were previously resurrected from a dated sediment archive of Lake Ring (Jutland, Denmark; 55°57′51.83″ N, 9°35′46.87″ E), a shallow lake with a century‐long, well‐documented record of anthropogenic impact (Cuenca Cambronero et al. 2018; Eastwood et al. 2023). The lake was semi‐pristine between the 1900s and 1950s (semi‐pristine phase; SP). Following the discharge of sewage from a nearby town, the lake's trophic level increased, resulting in eutrophication between the 1950s and the 1970s (eutrophic phase; EP). The sewage inflow was diverted in the late 1970s, but this coincided with an increase in pesticide run‐off from agricultural land surrounding the lake (1980–1990; pesticide phase; PP). In recent times, the decline in agricultural land use led to a partial recovery of the lake (recovery phase; RP) (Cuenca Cambronero et al. 2018; Davidson et al. 2007; Eastwood et al. 2023; Gigl et al. 2024).

Resurrected dormant *D. magna* genotypes are the results of sexual reproduction and are genetically unique. Once hatched, each genotype can be propagated indefinitely under laboratory conditions via parthenogenetic reproduction, enabling the use of clonal replicates across experiments. We selected four genotypes per population, each corresponding to a lake phase (total = 16 genotypes), and exposed them to 428 μg/L of phenanthrene (PHE) across their full life cycle. This concentration corresponds to the median EC₁₀ derived from acute toxicity assays across all genotypes (Gigl et al. 2024). Previous whole‐genome analyses of resurrected *Daphnia* populations have shown that high standing genetic variation and large effective population size allow a small number of genotypes to capture most population‐level genetic diversity, even under strong selection (Chaturvedi et al. 2021). Based on this evidence, four genotypes per lake phase were sampled, as this level of replication is sufficient to represent standing genetic variation while enabling feasible transcriptomic and microbiome analyses. The temporal subpopulations analysed across lake phases originate from the same underlying genetic pool, maintained through a large dormant egg bank. Dormant egg banks in *Daphnia* have been shown to represent an unbiased sample of population genetic diversity and evolutionary potential, with no evidence that dormancy duration systematically biases functional traits (Orsini et al. 2016). These features support the robustness of our experimental design, while maintaining feasibility.

Full exposure protocols are detailed in Gigl et al. (2024). In brief, genotypes were acclimated for two generations at 20°C ± 1°C under a 16:8 h light–dark cycle and fed *ad libitum* with 0.8 mg/L *Chlorella vulgaris* (strain CCAP 211/11B) to minimise interference from maternal effects. After acclimation, 24‐h‐old neonates from the second or subsequent broods were randomly assigned to experimental exposures. Life history traits were analysed as described in Gigl et al. (2024). In parallel, *Daphnia* tissue was sampled from clonal replicates for transcriptome and microbiome profiling, which forms the focus of the present study. Both transcriptomic and gut microbiome data were obtained from whole *Daphnia*, using a split‐sample design in which pools of at least ten individuals from the same biological replicate were assigned to RNA and DNA extractions. Together, these datasets provide complementary insights into the effects of PHE on the health and resilience of a sentinel aquatic species. Tissue samples were collected at the final juvenile instar, prior to the onset of sexual maturation, to minimise confounding effects associated with reproductive development. For each genotype, parallel control replicates (unexposed to PHE) were included to enable comparative analysis. At the end of the exposure period, individuals were gently removed from the medium, blotted to remove excess water, and immediately flash‐frozen in liquid nitrogen. All samples were stored at −80°C until further processing for RNA and DNA extraction.

### 2.2 *Daphnia* Transcriptome

Flash‐frozen tissue from experimental exposures was homogenised for total RNA extraction with a 2020 Geno/Grinder (Cole‐Parmer Simpleprep) and by using the Agencourt RNAdvance Tissue Total RNA kit (Beckman Coulter). The concentration and the quality (integrity and purity) of the extracted RNA were quantified with the Nanodrop 8000 Spectrophotometer (Labtech Ltd., U.K.) and the Bioptic Qsep 100 Advance using Bioptic HS‐RNA Cartridges (C105111). The mRNA was prepared into cDNA libraries using the NEBNext Ultra Directional RNA Library Prep Kit (New England Biolab E7420L) with NEBnext Multiplex Oligos for Illumina Dual Index Primers (New England Biolabs E7600S). RNA extraction and library preparation were performed with the Biomek FxP workstation (Beckman Coulter A31842). The library's quality was assessed using Bioptic Qsep 100 Advance. Libraries were pooled and 150‐bp PE sequenced on a DNB Seq‐G400 platform at the Beijing Genomics Institute (BGI), aiming for 5 million reads per sample. RNA extraction and cDNA libraries were prepared by EnviSion, BioSequencing and BioComputing at the University of Birmingham, UK.

### 2.3 *Daphnia* Gut Microbiome Response to PHE

Bacterial DNA for all exposures was extracted from flash‐frozen tissue, using the QIAamp DNA Microbiome Kit (Qiagen 51,704) in a PCR free environment. Tissue was homogenised with a 2020 Geno/Grinder (Cole‐Parmer Simpleprep). Genomic DNA quality and concentration were assessed using a Bioptic Qsep 100 Advance with Bioptic High Sensitivity Kit (C105105). Paired end 300 bp amplicon libraries were prepared for the 16SV1 (fwd—AGAGTTTGATCMTGGCTCAG, rev—TGCTGCCTCCCGTAGGAG (Mao et al. 2012)) and 16SV4 (fwd—GTGCCAGCMGCCGCGGTAA, rev‐ GGACTACHVGGGTWCTAAT (Caporaso et al. 2011)) regions following a standard 2‐step PCR protocol with the Qiagen Multiplex Master Mix. This protocol includes a primary reaction for the target marker gene regions in PCR1, in which primers with 5′ sequence overhangs are added to the marker gene of choice, and a PCR2, which ligates sequencing adapters and indices to the cleaned PCR1 products (Eastwood et al. 2024; Ushio et al. 2022). Triplicate samples were amplified in PCR1 using Q5 HS High‐Fidelity Master Mix (New England Biolabs) following the manufacturer's instructions. PCR2 amplicons were mixed in equimolar quantities (at a final concentration of 12 pmol) using a Biomek FXp l workstation (Beckman Coulter A31842). Pool's molarity was confirmed using the Qsep 100 Advance (AutoQ Biosciences) with High Sensitivity DNA cartridges (AutoQ Biosciences C105105) prior to PE 300 bp sequencing on a DNB‐Seq‐G400 platform at BGI. We aimed for 100,000 reads per sample and biological replicate. DNA extraction and metabarcoding libraries were prepared by EnviSion, BioSequencing and BioComputing at the University of Birmingham, UK.

### 2.4 Bioinformatics Analysis

#### 2.4.1 Data Quality and Preliminary Analyses

##### 2.4.1.1 Transcriptome

The quality of trimmed RNA‐seq reads was assessed using FastQC (version 0.11.9) (Andrews 2010; Wingett and Andrews 2018) complemented by additional quality metrics from alignment statistics obtained through Qualimap (García‐Alcalde et al. 2012) and STAR (Dobin et al. 2013). Reads failing quality criteria established by FastQC and Qualimap were excluded from downstream analysis. The retained high‐quality reads were aligned to the *D. magna* reference genome (Chaturvedi et al. 2023) using the alignment software STAR (version 2.7.11a‐GCC‐12.2.0) (Dobin et al. 2013). Post‐alignment, genes were counted using HTSeq (Anders et al. 2015). Differential gene expression analysis was performed using DESeq2 (Love et al. 2014). Prior to differential expression analysis, we normalised read counts and explored patterns of gene expression, similarity and divergence across samples using multiple dimensionality reduction techniques, for example, principal component analysis (PCA). To enhance statistical robustness, genes with cumulative expression counts smaller than 10 across samples were filtered out prior to downstream analysis. Differentially expressed genes were identified based on *p*‐values ≤ 0.05.

##### 2.4.1.2 Microbiome

Raw sequencing reads were demultiplexed using the forward PCR1 primer sequence with Cutadapt v3.7.4, allowing for one mismatch (error rate = 0.07). Sequence quality was then assessed using FASTQC (Wingett and Andrews 2018) and summarised with MultiQC (Ewels et al. 2016). High‐quality sequences were imported into QIIME2 v2021.2 (Bolyen et al. 2019), where they were trimmed, quality‐filtered, merged, and denoised using the DADA2 plugin (Callahan et al. 2016) with default parameters. Primer sequences and low‐quality regions were removed during this step.

Taxonomic classification was performed using a Naive Bayes classifier trained on the SILVA v138 reference database (Quast et al. 2012). Taxonomy was assigned using the *classify‐sklearn method* from the QIIME2 feature‐classifier plugin and analysed at both the genus and family levels (Pedregosa et al. 2011). Denoised amplicon sequence variant (ASV) tables were exported to R (v4.4.1) and processed using the *phyloseq* package (McMurdie and Holmes 2013). Samples with fewer than 5000 reads and taxa with fewer than 10 total reads or present in less than 5% of samples were removed using the *phyloseq_filter_prevalence* function from *metagMisc* v0.5.0 (https://github.com/vmikk/metagMisc/tree/0.5.0). Retained samples were rarefied to the minimum sequencing depth of each dataset (16S V1: 148,021; 16S V4: 82,189) using *rarefy_even_depth* from *phyloseq*, with replacement and a fixed random seed to ensure reproducibility (Weiss et al. 2017) For 16S V1, sample LRV9.5_3_PHE1 (Recovery, treated) was excluded during rarefaction due to insufficient reads. For 16S V4, sample LRII_44_8.1_C3 was excluded post‐rarefaction. Good's coverage exceeded 99.9% across rarefied samples (median = 1.00; Good 1953).

Shannon diversity was calculated using *estimate_richness* from phyloseq. Outliers were identified within each population (Semi‐Pristine, Eutrophic, Pesticide, Recovery) and treatment group (control, treated) using the interquartile range (IQR) method (values outside Q1–1.5 × IQR or Q3 + 1.5 × IQR). For 16S V1, the following outliers were removed: LRII_88_1 (control); LRV12.4 (control) and LRV12.5_1 (PHE exposed); and LRV2.1 and LRV2.5_9 (both PHE exposed); and PHE2 (Recovery, treated). For 16S V4, one outlier was detected: LRV12.5 (PHE exposed) and excluded from downstream analysis. Wilcoxon rank‐sum tests were used to compare Shannon diversity between control and treated samples within each lake phase. *p*‐values were adjusted for multiple comparisons. Boxplots were generated using the ggplot2 and ggpattern (Wickham and Sievert 2009).

To assess differences in microbial community composition, we performed a permutation‐based multivariate analysis of variance (PERMANOVA), following Anderson (2014). A nested experimental design was applied to account for fixed effects (treatment and population) and random effects (biological replicates). Bray–Curtis dissimilarity matrices were computed from rarefied ASV tables, and 999 permutations were conducted. To preserve the hierarchical structure of the dataset, permutations were restricted within populations, preventing randomization across groups with distinct baseline communities.

A Type III sum‐of‐squares model was used to evaluate main effects and interaction terms between treatment and population. To further investigate specific differences, we performed pairwise *post hoc* PERMANOVA tests between populations and between control and treatment samples using a custom R script. This script was used to subset the Bray–Curtis matrix, exclude groups with fewer than six samples, run a PERMANOVA with 999 permutations, and apply a Benjamini–Hochberg false discovery rate (FDR) correction to control for multiple comparisons (Benjamini and Hochberg 1995).

#### 2.4.2 Functional Analysis

##### 2.4.2.1 Transcriptome

The differentially expressed genes (DEGs) were subjected to pathway overrepresentation analysis using the Reactome Pathway Analysis Toolkit (Milacic et al. 2024). Reactome is a manually curated and peer‐reviewed database of biological pathways that maps gene functions across a wide range of processes. Although primarily based on human data, Reactome incorporates orthology‐based predictions to extend coverage to 14 additional non‐human species, enabling cross‐species functional annotation. The analysis identified significantly enriched pathways (*p* ≤ 0.05).

In parallel, we conducted pathway enrichment analysis using the Kyoto Encyclopedia of Genes and Genomes (KEGG) database (Kanehisa et al. 2025; Milacic et al. 2024). DEGs were mapped to KEGG pathways, and enrichment was evaluated using a Fisher's exact test, followed by adjustment for multiple testing. Although KEGG offers less extensive coverage of eukaryotic signalling pathways compared to Reactome, it excels in representing entire biochemical systems, particularly metabolic and enzymatic pathways, through a combination of manual curation and computational annotation. Pathways with adjusted *p*‐values (*p*_adj_ ≤ 0.05) were considered significantly enriched.

The combined use of Reactome and KEGG databases allowed for complementary insights into gene expression changes, enabling a more robust characterisation of the biological processes and molecular functions affected by phenanthrene exposure.

##### 2.4.2.2 Microbiome

Differential abundance analysis of microbial communities across populations from different lake phases was performed using the DESeq2 package (Love et al. 2014) in R v4.4.1. Prior to analysis, amplicon sequence variants (ASVs) were pre‐processed by removing features with zero counts across all samples and filtering out low‐abundance ASVs, defined as those present in ≤ 3 samples or with total counts ≤ 10. Following this filtering step, DESeq2 was applied to the ASV count data to identify differentially abundant taxa across experimental groups. The DESeq2 model included population (Eutrophic, Pesticide, Recovery, Semi‐Pristine) and treatment (PHE exposure vs. control) as fixed effects. Size factors for normalisation were estimated using the *poscounts* method, which is recommended for sparse microbiome data (McMurdie and Holmes 2014). Differential abundance was assessed at both the ASV level and the level of predicted functional categories, with statistical significance determined using Benjamini‐Hochberg false discovery rate (FDR) correction (Benjamini and Hochberg 1995), applying an adjusted *p*‐value threshold of ≤ 0.05.

To explore microbial community function, functional profiles were predicted using PICRUSt2 (Douglas et al. 2020), which infers metagenomic content from 16S rRNA gene sequences. Predicted KEGG Orthology (KO) abundances were generated for each genotype and treatment condition, harmonised with the filtered sample set, and analysed using DESeq2 to identify differentially abundant KOs across treatments and populations.

Pathway enrichment analysis of the differentially abundant KO terms was then conducted using over‐representation analysis (ORA) via the *enrichKEGG* function in the *clusterProfiler* package (Yu et al. 2012). This method applies a one‐sided Fisher's exact test to identify KEGG pathways significantly over‐represented among the differentially abundant KO sets. Pathways with FDR‐adjusted *p*‐values ≤ 0.05 and *q*‐values ≤ 0.2 were considered significantly enriched.

## 3 Results

### 3.1 *Daphnia* Transcriptional Response to PHE

We quantified the genome‐wide transcriptional response of *Daphnia* temporal populations to PHE (NCBI BioProject PRJNA1289021). Using exploratory analysis tools such as PCA, we found that treatment was the primary driver of variation in transcriptional responses across the dataset. Although separation by population was not consistent across all phases, the semi‐pristine population emerged as more distinct compared to the others (Figure S1). The first three PC components explained 52% of the overall variance. This suggests that other factors, such as genotype, likely contribute to the overall variance.

**FIGURE 1:**
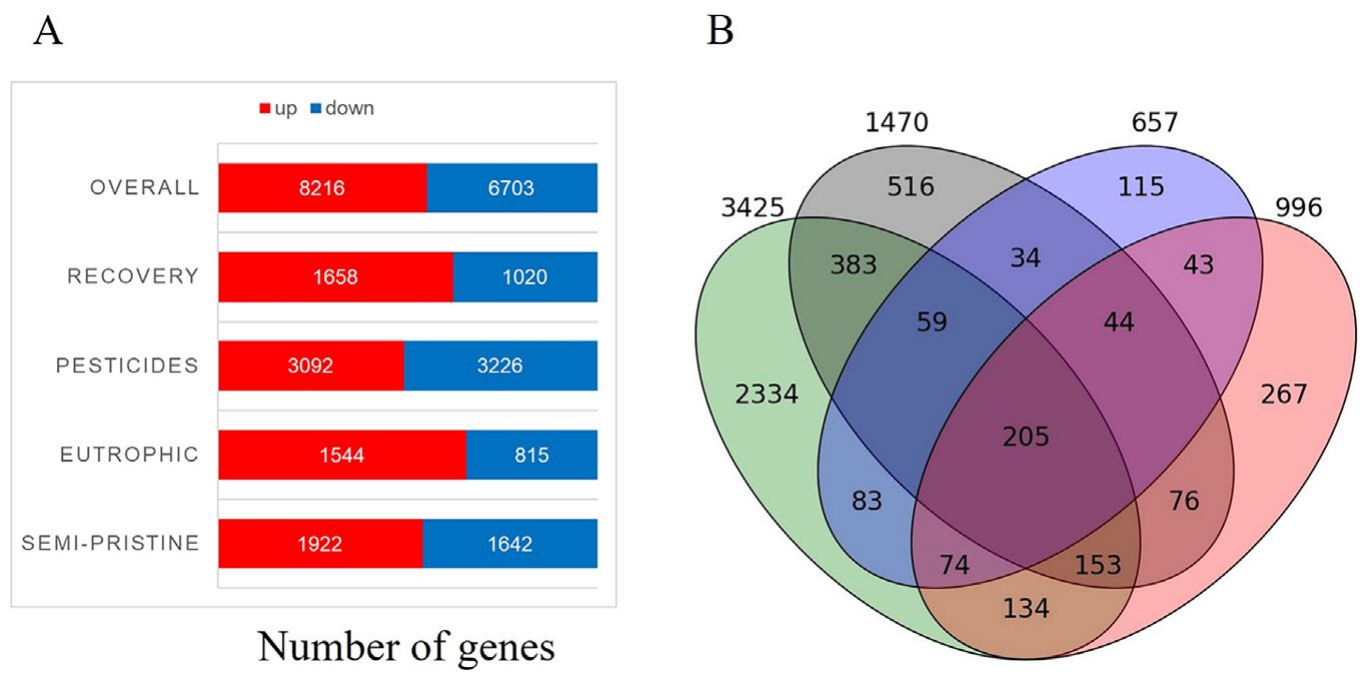
Differentially expressed genes. (A) Differentially expressed genes identified across the dataset and shown separately for each population (corresponding to lake phases). The genes are categorised as upregulated (ref) or downregulated (blue); (B) differentially expressed genes under PHE treatment shared among the four *Daphnia* populations. The Read count table to support the plots is in Table S1.

Across the four *Daphnia* populations studied here, we identified 14,919 differentially expressed genes, of which 8216 were up and 6703 were downregulated (Figure 1A). The lower number of differentially expressed genes was identified in the eutrophic population (2,359 genes), followed by the recovery (2,678 genes) and semi‐pristine populations (3,564 genes), with the pesticide population showing the largest number of differentially expressed genes (6,318 genes) (Figure 1A; Table S1). The differentially expressed genes responding to PHE exposures and unique to each population of *Daphnia* were as follows: Semi‐Pristine: 516 genes; Eutrophic: 115 genes; Pesticide: 2334 genes; and Recovery: 267 unique genes. The genes shared between each pair of populations ranged between 800 (Pesticide and Semi‐pristine populations) and 366 (Eutrophic and Recovery populations) (Figure 1B; Table S1).

### 3.2 Functional Changes in *Daphnia* Transcriptome Induced by PHE

Differentially expressed genes were mapped onto pathways using both the Reactome and KEGG databases for a better understanding of the functional divergence among populations. Reactome returned a substantially higher number of pathways than KEGG, reflecting its more detailed and fine‐grained annotations. KEGG groups biological processes into broader categories, resulting in fewer, more generalised pathway assignments.

**FIGURE 2:**
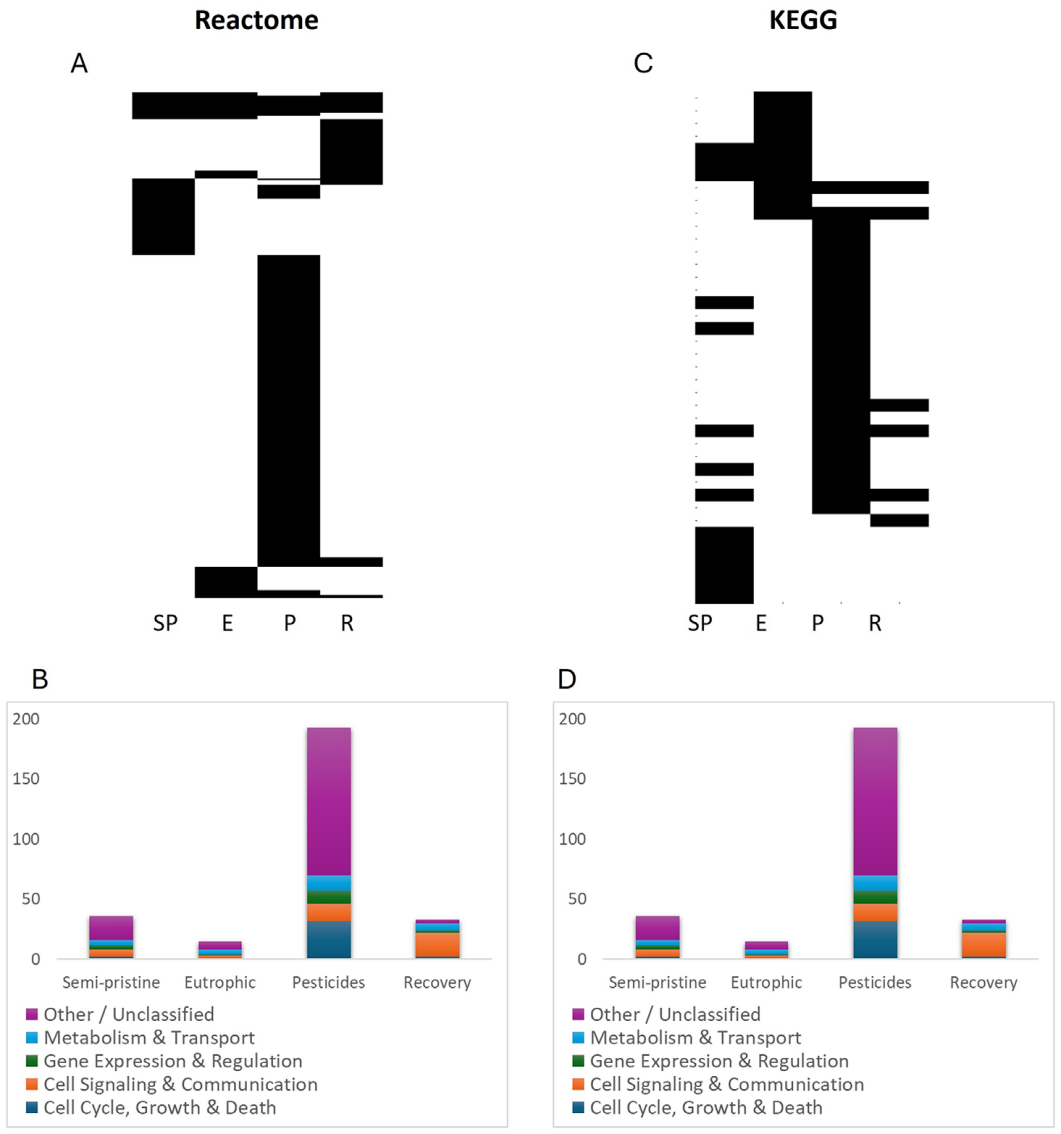
Pathways in the *Daphnia* populations. Overview of pathways presence/absence in the *Daphnia* populations identified via the overrepresentation analysis within the Reactome database (A) and the enrichment analysis within the KEGG database (B). The black colour indicates the presence of a pathway, whereas the white colour indicates the absence of a certain pathway in the specific population. The population names are abbreviated as follows: SP, Semi‐pristine; E, Eutrophic; P, Pesticides; R, recovery. The top XX pathways showing a *p*‐value < 0.05 and that were shared across *Daphnia* populations are shown for the Reactome (C) and the KEGG database (D). Panels C and D are supported by Table S2.

A total of 323 non‐redundant pathways were identified in the Reactome database. Of these, 277 pathways were unique to a single population (Semi‐pristine: 36; Eutrophic: 15; Pesticides: 193; Recovery: 33) and 46 were shared among two or more populations (Figure 2A; Table S2). The pathways shared across multiple populations were enriched in core cellular processes such as cytoskeleton organisation, motility, and gene expression (Figure 2B; Table S2). At a finer level of classification, these included conserved elements of GTPase cycles, RNA processing, and second messenger signalling, underscoring their central role in maintaining cellular structure, information flow, and intracellular communication across wild *Daphnia* populations.

Pathways enriched in specific populations reflected divergent physiological adaptations shaped by their exposure histories to pollution. The Semi‐pristine and Eutrophic populations exhibited relatively few unique pathways, which were more evenly distributed across functional categories (Figure 2B; Table S2). In the Semi‐pristine population, these included processes related to gene expression machinery, such as transcriptional regulation by MECP2 and RNA polymerase activity, alongside cell adhesion and extracellular matrix organisation, suggesting a stable and regulated physiological state. The Eutrophic population displayed modest enrichment in metabolic and detoxification pathways, such as fatty acid metabolism and oxidative catabolism, potentially linked to increased metabolic turnover in nutrient‐enriched conditions (Figure 2B; Table S2). In contrast, the Pesticide population exhibited a substantially higher number of unique pathways than all the other populations, with a pronounced bias toward cell cycle regulation, DNA damage repair, and cell survival mechanisms. These included pathways governing mitotic checkpoints, oxidative stress responses, and autophagic clearance, reflecting a broad activation of cellular defence and recovery programmes under chronic toxicant exposure (Figure 2B; Table S2). Finally, the Recovery population showed enrichment in cell signalling and immune response pathways, including MAP kinase cascades, interleukin signalling, and growth factor receptor pathways. These signatures indicate a shift from acute stress response toward immune recalibration and tissue regeneration during recovery from previous pesticide exposure (Figure 2B; Table S2).

A total of 40 non‐redundant pathways were identified in the KEGG database. Of these, 29 were unique to a single population (Semi‐pristine: 6; Eutrophic: 5; Pesticide: 17; Recovery: 1), and 11 were shared among two or more populations (Figure 2C; Table S2). Shared pathways primarily clustered within cytoskeleton and motility, and GPCR and signalling, reflecting conserved physiological functions such as intracellular trafficking, cell structure maintenance, and environmental sensing (Figure 2D; Table S2). In the Semi‐pristine population, unique pathways spanned categories such as gene expression and regulation (e.g., transcriptional regulation by MECP2), cell adhesion and extracellular matrix (e.g., integrin interactions), metabolism and detoxification (e.g., cytosolic sulfonation), suggesting preserved regulatory control and moderate detoxification activity under low‐stress conditions. The Eutrophic population showed modest but broad representation across signalling (e.g., GTPase cycles), metabolic (e.g., fatty acid oxidation), and gene expression processes, consistent with the increased metabolic demand typical of nutrient‐rich environments (Figure 2D; Table S2). The Pesticide population exhibited the highest number of unique pathways, with strong enrichment in categories related to cell cycle and division, DNA damage and stress response, and cell death and survival. These included pathways for DNA repair, oxidative stress response, and autophagy, reflecting intensive cellular adaptation to sustained chemical exposure (Figure 2D; Table S2). In contrast, the Recovery population exhibited a narrower response, with five unique pathways including tryptophan metabolism and MAP kinase signalling. These mapped to immune signalling, neurological and sensory processing, suggesting a post‐stress shift toward immune recalibration and neuroendocrine recovery (Figure 2D; Table S2).

At the genotype level, Reactome pathway analysis revealed a broad functional landscape, with most enriched pathways falling into categories related to signalling networks, metabolism and biosynthesis, and gene expression machinery (Figure S2A; Table S2). Analysis of KEGG pathways across *Daphnia* genotypes revealed that most detected pathways fell under broad categories of lipid metabolism, transport processes, and biosynthetic functions, with additional representation from glycan metabolism, signalling, and energy metabolism (Figure S2B; Table S2). These functions were consistently represented across genotypes, suggesting that core physiological processes such as transcriptional control, intracellular signalling, and metabolic regulation are tightly conserved but variably activated among individual *Daphnia* genotypes. Additional pathway enrichment at the genotype level was observed in cell cycle regulation, DNA damage and repair, and cell death and survival in the Reactome analysis, whereas the KEGG analysis showed prevalence of lipid and glycan metabolism pathways suggesting genotype‐specific variation in membrane structure and energy storage (Table S2). While the consistent presence of transport and signalling pathways highlights conserved mechanisms for environmental sensing and molecular trafficking, the results also indicate genotype‐specific activation of stress response mechanisms. These results reinforce population‐level findings, suggesting that underlying genetic variation contributes to the differential deployment of functional responses across environmental gradients. A substantial proportion of pathways remained unclassified, likely reflecting diverse or poorly annotated molecular functions (Table S2).

### 3.3 *Daphnia* Gut Microbiome Response to PHE

We assessed the impact of PHE exposure on gut microbiota composition and diversity in the same *Daphnia* populations analysed for transcriptional responses (NCBI BioProject PRJNA1289019).

**FIGURE 3:**
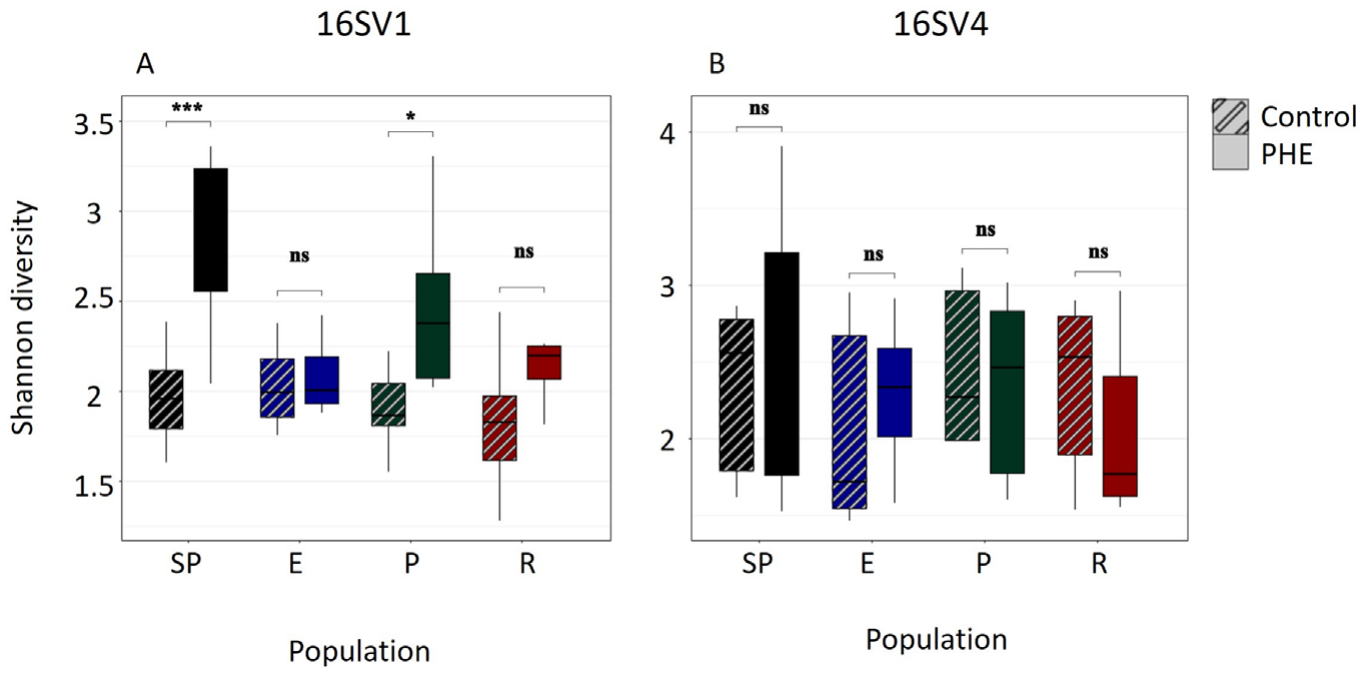
Alpha diversity. Alpha diversity, measured as Shannon entropy, between control (dashed pattern) and treated (solid pattern) *Daphnia* population, as measured for the 16SV1 (A) and 16SV4 (B) marker genes. These plots are supported by paired Wilcoxon tests and significant pairwise differences indicated (*p* < 0.05). ns, non‐significant. The population names are abbreviated as in Figure 1.

Alpha diversity, a measure of within‐sample microbial diversity, was quantified using the Shannon entropy index. For the 16SV1 marker gene, a significant increase in alpha diversity was observed in PHE‐exposed individuals compared to controls in the Semi‐Pristine (*p* = 0.00005) and Pesticide (*p* = 0.05) populations (Figure 3A). In contrast, no significant differences were detected in the Eutrophic (*p* = 0.463) and Recovery (*p* = 0.257) populations, suggesting either resilience or pre‐existing community alterations that buffer against this disturbance. For the 16SV4 marker gene, alpha diversity did not differ significantly between PHE‐exposed and control samples across the four populations: Semi‐Pristine (*p* = 0.401), Eutrophic (*p* = 0.606), Pesticide (*p* = 0.662), and Recovery (*p* = 0.589) (Figure 3B). These contrasting patterns between marker regions can be expected as they target different parts of the prokaryotic gut community.

**FIGURE 4:**
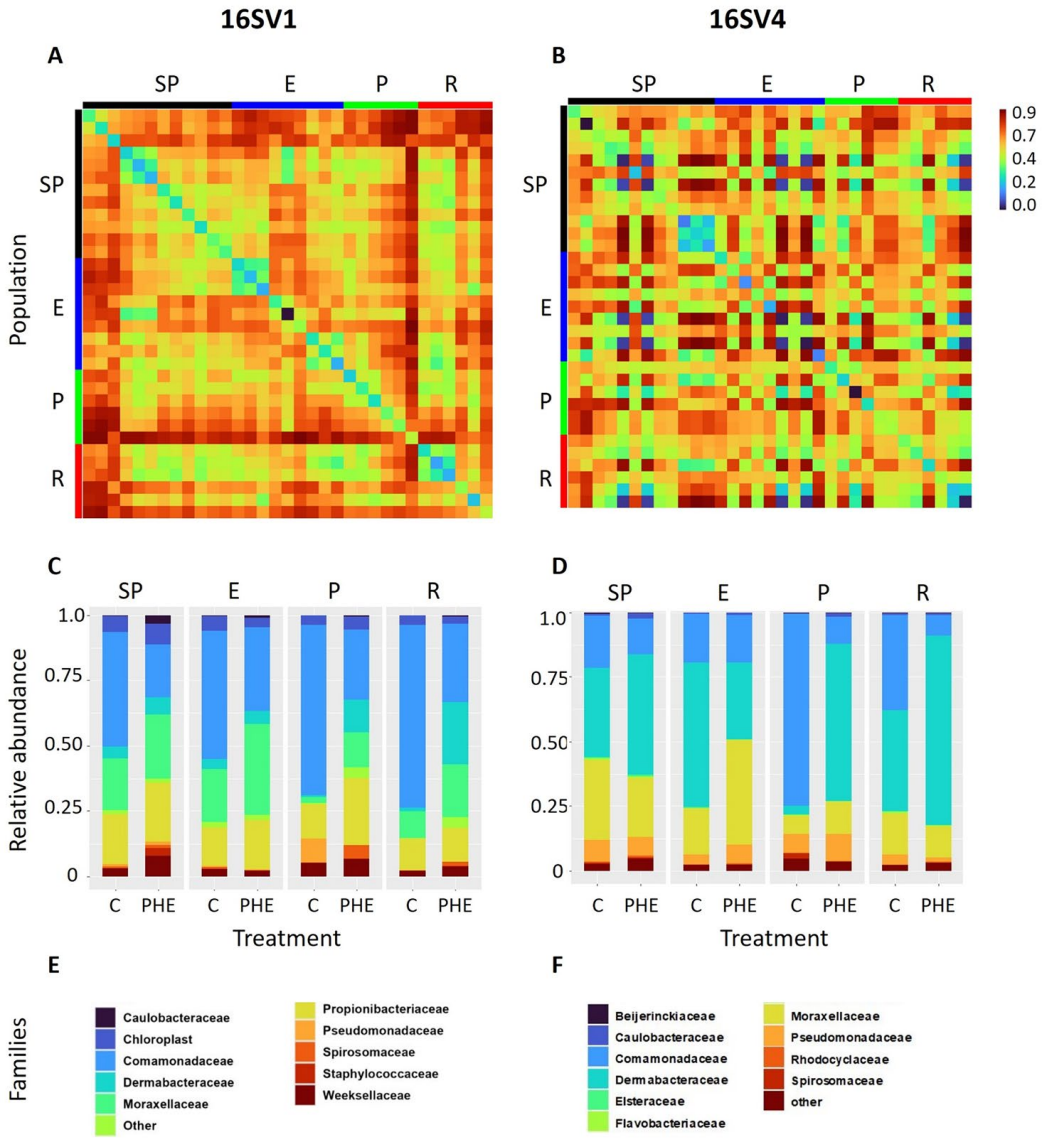
Microbial community composition. (A, B) Heatmaps showing community dissimilarity (Beta diversity) between each pair of populations, measured as Bray–Curtis; (C, D) Relative abundance of the top 11 bacterial families detected in non‐exposed (C‐control) and PHE‐exposed (PHE) populations for 16SV1 and 16SV4 gene markers; (E, F) most abundant families. The colours are the same as in C and D. Colours are specific to each plot; the same colour may represent different bacterial families across the two marker genes. The supporting statistics for this figure are in Table 1. The population names are abbreviated as in Figure 1.

Beta diversity analysis revealed that microbial community composition significantly differed between PHE‐exposed and control samples for the 16S V1 marker gene (Table 1; Treatment), as well as among *Daphnia* populations representing different lake phases (Figure 4A,B; Table 1). However, the interaction between treatment and population was not statistically significant, indicating that the effect of PHE exposure on microbial composition did not vary significantly across populations. Post hoc analysis on the 16S V1 revealed that the observed differences in microbial communities across populations were primarily driven by significant differences between Semi‐pristine and Pesticides populations (*p* = 0.042), and between Eutrophic and Pesticides populations (*p* = 0.054) (Table S3). The *post hoc* analysis confirmed that PHE‐exposed samples differed significantly from the respective control in all pairwise comparisons, except for the Eutrophic population (Semi‐pristine—*p* = 0.016; Pesticides—*p* = 0.026; and Recovery—*p* = 0.041). Analysis based on the 16S V4 marker gene showed no significant differences in beta diversity between PHE‐exposed and control groups, across populations, or for the treatment × population interaction (Figure 4A,B; Table 1). This was confirmed by the *post hoc* analysis (Table S3).

**TABLE 1:** PERMANOVA. Permutational Multivariate Analysis of Variance using Bray‐Curtis distances testing for significant differences among populations from different lake phases, treatment and their interaction genotypes at the two gene markers (16SV1 and 16SV4) with 999 permutations.

Effect	16SV1			16SV4		
	Df	*F*‐statistics	*p*	Df	*F*‐statistics	*p*
PHE	1	5.97	**0.001**	1	1.8	0.17
Population	3	2.3	**0.001**	3	1.34	0.07
PHE*Population	3	1.53	0.15	3	1.78	0.14

*Note:* Significant terms (*p*‐values < 0.05 after applying Benjamini & Hochberg correction for multiple testing) are in bold.

The topmost abundant families identified in the *Daphnia* gut microbiome using the 16SV1 were: *Caulobacteraceae*, *Comamonadaceae*, *Dermabacteraceae*, *Moraxellaceae*, *Propionibacteriaceae*, *Pseudomonadaceae*, *Spirosomaceae*, *Staphylococcaceae*, and *Weeksellaceae* (Figure 4C,E). The top‐most abundant families identified by the 16SV4 gene marker were: *Beijerinckiaceae*, *Caulobacteraceae*, *Comamonadaceae*, *Dermabacteraceae*, *Elsteraceae* (formerly known as *Azospirillaceae*), *Flavobacteriaceae*, *Moraxellaceae*, *Pseudomonadaceae*, *Spirosomaceae*, and *Rhodocyclaceae* (Figure 4D,F). While these bacteria families were shared across all populations and between control and exposed populations, their relative abundances shifted significantly in the populations exposed to PHE and among populations, as shown by the beta diversity supported by the PERMANOVA analysis. For completeness, we also report the most abundant genera identified in the PHE‐exposed and control populations across the two gene markers (Figure S3).

### 3.4 Functional Changes in *Daphnia* Gut Microbiota Induced by PHE

**FIGURE 5:**
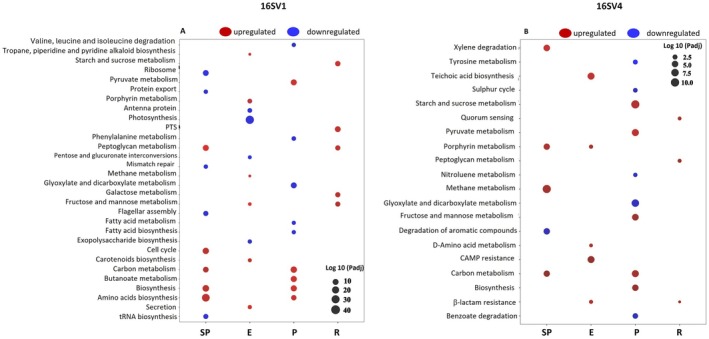
Enriched topmost abundant pathways in the gut microbial community. Topmost predicted microbial functional pathways in the *Daphnia* gut microbiome inferred from the 16SV1 (A) and 16SV4 (B) marker genes. Functional prediction was performed using PICRUSt2, and pathway over‐representation analysis was conducted to identify microbial functions significantly associated with PHE exposure across *Daphnia* populations, represented on the *x*‐axis: SP (Semi‐Pristine), E (Eutrophic), P (Pesticide), and R (Recovery). The *y*‐axis lists the topmost KEGG pathways predicted to be significantly enriched. Circles indicate pathways that are upregulated (red) or downregulated (blue) in PHE‐exposed samples relative to controls. Circle size is scaled to the −log10 (FDR‐adjusted *p*‐value), reflecting the strength of statistical significance in pathway enrichment. The complete list of enriched pathways in the *Daphnia* gut microbiota can be found in Table S4.

The observed shifts in community composition corresponded to different functions, as revealed by the pathway analysis. Pathway enrichment analysis revealed both overlapping and distinct functional profiles in microbial communities predicted using the 16S V1 and V4 marker regions. A total of 54 pathways were shared between regions, while 55 were unique to V1 and 6 to V4, indicating a higher functional resolution captured by the V1 region (Figure 5; Table S4).

The V1 marker region showed a diverse range of enriched pathways, particularly within metabolism‐related functions, with 194 pathways classified under this category (Figure 5A; Table S4). The predicted functional profiling of 16SV1‐associated communities revealed significant enrichment in butanoate metabolism, biosynthesis of amino acids, carbon metabolism, ribosome function, pyruvate metabolism, and the phosphotransferase system. This suggests active modulation of both core and specialised microbial metabolic processes, likely reflecting shifts in microbial energy production, nutrient utilisation, and translational machinery under PHE exposure. Additionally, the V1 marker gene captured pathways associated with cellular processes (e.g., cell cycle), genetic information processing (e.g., translation), and environmental information processing, indicating broader functional adaptations beyond basic metabolism (Figure 5A; Table S4).

The 16SV4 region identified a narrower set of predicted enriched pathways, with most falling under the metabolism category (62 pathways total—Figure 5B; Table S4), and a small number associated with disease, organismal systems, and cellular processes. Key functions enriched within the V4‐derived profiles included xylene degradation, teichoic acid biosynthesis, starch and sucrose metabolism, pyruvate metabolism, porphyrin metabolism, methane metabolism, glyoxylate and dicarboxylate metabolism, biosynthesis, and benzoate degradation (Figure 5B; Table S4). It is noteworthy the enrichment of the degradation of aromatic compound pathway in the 16SV4 region (Figure 5B; Table S4). These results suggest that the 16SV4 captures specific degradation and biosynthetic processes relevant to microbial environmental response, albeit with reduced coverage of regulatory or information‐processing pathways.

Furthermore, both marker genes consistently revealed enrichment in carbon metabolism and pyruvate metabolism, highlighting conserved microbial responses related to central carbon processing across both marker regions (Figure 5; Table S4).

## 4 Discussion

Understanding how organisms and their associated microbiota respond to environmental contaminants is critical for predicting ecological impacts and assessing population resilience. Despite their role in modulating host physiology, immunity, and stress responses, microbial communities—particularly gut microbiota—remain underrepresented in ecotoxicological studies (Chaturvedi et al. 2023; Li et al. 2021), but see (Yu et al. 2012) for a pioneer study including both host and microbiome analysis in ecotoxicology.

In this study, we bridge this knowledge gap by jointly analysing host transcriptomic and gut microbiome responses in *D. magna* exposed to sub‐lethal concentrations of PHE. Our results show that both host gene expression and predicted microbial functional profiles are shaped by PAH chemical stress, compounded by legacy exposure to anthropogenic pollutants, highlighting the value of integrated multi‐omics approaches for capturing the full biological response to pollution. In our study, resting egg age is necessarily correlated with lake phase. However, dormant egg banks in *Daphnia* have been shown to provide an unbiased representation of population genetic diversity and evolutionary potential (Orsini et al. 2016). This evidence, combined with acclimation for two clonal generations before the experimental exposures, is expected to minimise any residual carry‐over effects of diapause on transcriptional or microbiome responses (Orsini et al. 2016).

### 4.1 Host Transcriptional Response to PHE Exposure

PHE triggered widespread transcriptional reprogramming in *D. magna* , affecting key biological processes such as growth regulation, development, cytoskeletal organisation, and xenobiotic metabolism. These changes indicate a systemic physiological response to this hydrocarbon common in freshwater ecosystems.

Pathways involved in cuticle synthesis and chitin metabolism, critical for exoskeletal integrity and the moulting process, were significantly differentially enriched in PHE‐exposed populations. Such effects, reflecting plastic regulatory responses, have previously been reported in *Daphnia* and other aquatic invertebrates following exposure to PAHs, including benzo[*a*]pyrene and phenanthrene, which interfere with the timing and regulation of ecdysis by impairing structural gene expression and hormonal pathways controlling development (Cuvillier‐Hot and Lenoir 2020; Shatilina et al. 2020). Supporting previous literature on endocrine disruption by hydrocarbons, PHE exposure in *Daphnia* altered endocrine function, including dysregulation of ecdysteroid and juvenile hormone signalling pathways, which are central to reproduction and maturation. These hormonal disruptions likely contribute to the previously observed reproductive delays and reduced fecundity under PHE exposure reported for these same *Daphnia* populations in Gigl et al. (2024). We also observed significant upregulation of oxidative stress response genes, including multiple isoforms of cytochrome P450 monooxygenases (CYPs) and glutathione S‐transferases (GSTs), key players in phase I and phase II detoxification pathways. These enzymes catalyse the biotransformation and conjugation of xenobiotics, facilitating their excretion from the organism. Their upregulation suggests activation of canonical detoxification mechanisms in *Daphnia* in response to aromatic hydrocarbon toxicity, confirming previously observed mechanisms in both invertebrates and vertebrates (Lemgruber et al. 2013; Nota et al. 2009; Yadetie et al. 2022).

### 4.2 Gut Microbiota Functional Shifts in Response to PHE

Significant increases in alpha diversity following PHE exposure in the Semi‐pristine and Pesticides populations in the 16SV1 marker gene suggest that previously unexposed and historically exposed microbiomes to anthropogenic pollutants derived from land use and industrialisation may respond to acute exposure to toxicants by increasing taxonomic richness and evenness. Whereas numerous studies have demonstrated that *D. magna* microbiome is genetically inherited and varied among populations and genotypes (Choi et al. 2024; Frankel‐Bricker et al. 2020; Hegg et al. 2021; Suppa et al. 2020), increased gut microbiota diversity is generally associated with positive impacts on the organism's fitness and survival, especially in fluctuating environments. However, it has been shown that exposure to certain stressors increases *Daphnia* gut microbial diversity, particularly when the stressor disrupts stable communities and allows opportunistic or stress‐tolerant taxa to proliferate (Choi et al. 2024; Juma et al. 2020). In contrast, the absence of alpha diversity changes in the Eutrophic and Recovery populations may reflect either microbial resilience due to previous exposure to pollutants or reduced responsiveness due to already disturbed communities, as seen in long‐term eutrophic systems where microbial plasticity is diminished (Plath and Boersma 2001).

The gut community composition was significantly altered by PHE exposure, as evidenced by changes in Beta diversity. However, the two marker genes showed different patterns, and the treatment effect was compounded by population‐specific and historical environmental differences, which we discuss in the next session. The absence of significant changes in the V4 dataset, except for a marginal effect in the Pesticide population, highlights community‐specific sensitivity, likely due to the differential microbial fractions targeted by the V1 and V4 marker regions. This difference may reflect higher taxonomic resolution of the V1 regions for *Daphnia*‐associated gut taxa, whereas the V4 region shows more conserved community structure. This underscores the value of using multiple gene markers to enhance the resolution and informativeness of microbiome studies. Alternatively, employing full‐length 16S rRNA sequencing could provide even higher taxonomic and functional resolution (Johnson et al. 2019).

The population‐wide response to PHE compounded by population‐specific historical exposure to pollutants indicates that while all populations exhibit gut microbiome shifts in response to PHE, the magnitude and nature of these shifts are influenced by baseline community differences shaped by each population's environmental history and genetic background. Although dysbiosis following toxicant exposure is well documented across taxa, from vertebrates to invertebrates (Kaur and Rawal 2023; Suppa et al. 2020; Yan et al. 2020), our study uniquely separates constitutive (genetic) differences from plastic responses to acute chemical stress.

The compositional shifts in gut community composition observed in our study were accompanied by changes in predicted microbial functions. In the 16SV1 region, PHE exposure altered both core and specialised metabolic pathways, including butanoate and pyruvate metabolism, amino acid biosynthesis, carbon metabolism, ribosome function, and the phosphotransferase system, indicating adjustments in microbial energy production, nutrient utilisation, and translational processes. Additionally, the 16SV1 marker gene captured functional pathways associated with the cell cycle, translation, and environmental sensing, pointing to broader microbial adaptations that extend beyond primary metabolism.

Despite the absence of significant shifts in overall community composition in the 16SV4 dataset, changes in the relative abundance of specific bacterial groups led to enrichment of specialised degradation pathways, including those for xylene, benzoate, and methane metabolism. This was particularly evident in the Pesticides population, where the 16SV4 region captured enrichment in aromatic compound degradation pathways. These findings highlight the region's sensitivity to detecting microbial functions relevant to environmental response, despite its more limited resolution for regulatory and information‐processing pathways. The observed enrichment of antimicrobial resistance and membrane biosynthesis pathways, such as teichoic acid biosynthesis, suggests microbial adaptation strategies aimed at maintaining cell envelope integrity and function under chemical stress (Poole 2012). This response pattern is consistent with findings on invertebrates exposed to environmental toxicants, where the gut microbiome plays a central role in mediating host tolerance and resilience to pollutants (Diner et al. 2024).

Importantly, both marker regions showed consistent enrichment in central carbon metabolism and pyruvate metabolism pathways. These core functions likely represent universal microbial responses to PAH exposure, supporting community resilience and functional redundancy under chemical stress. Such metabolic plasticity is a conserved feature across animal‐associated microbiomes, enabling adaptation to external disturbances, including chemical pollution. This convergence on fundamental metabolic pathways suggests that, despite taxonomic and functional variation across populations and marker regions, the gut microbiome maintains essential functions that buffer the host against environmental stressors (Lee and Hase 2014; See et al. 2025).

### 4.3 Population‐Specific Functional Responses

Our analysis revealed differences in functional response to hydrocarbons among populations with different histories of exposure to pollution, challenging current paradigms and showing how historical exposure to chemical stress shapes response to chemical pollutants. These differences were evident in the host functional response as well as its associated gut microbiota.

In the Semi‐pristine population, pathway enrichment evidenced by Reactome analysis was primarily associated with transcriptional regulation mechanisms, including MECP2‐mediated control and RNA polymerase activity, as well as extracellular matrix organisation. These patterns suggest a relatively stable and tightly regulated physiological state under minimal stress conditions. In contrast, the Eutrophic population exhibited enrichment in pathways linked to fatty acid metabolism and oxidative catabolism. This finding aligns with literature showing that nutrient enrichment can elevate basal metabolic rates in freshwater zooplankton and shift energy allocation toward detoxification and maintenance processes (Anderson et al. 2004; Plath and Boersma 2001). In nutrient‐rich environments, *Daphnia* experience shifts in their energy budget and can be subjected to oxidative stress, particularly when food quality or quantity is high. While abundant food initially boosts energy reserves, it can later lead to increased sensitivity to other stressors and a shift in life history toward reproduction, potentially impacting the organism's ability to cope with additional challenges (Bergman Filho et al. 2011). The Pesticide population demonstrated the most extensive and functionally diverse transcriptional response, including enrichment in pathways governing cell cycle progression, DNA damage repair, oxidative stress responses, and autophagic clearance. These findings suggest a chronic engagement of cellular defence and repair mechanisms, consistent with prolonged toxicant exposure (Orsini et al. 2018; Osborne et al. 2007; Rothenberg et al. 2015). However, persistent activation of these pathways may carry metabolic costs and impair physiological plasticity, potentially leading to trade‐offs in growth or reproduction (Rauw 2012). The pesticide population showed the highest fitness costs in the study of Gigl et al. (2024), supporting this conclusion. Interestingly, the Recovery population, which is derived from historically pesticide‐contaminated habitats, exhibited pathway enrichment in MAP kinase signalling, cytokine‐mediated immune responses, and growth factor receptor pathways. These suggest a shift from acute stress signalling toward immune recalibration and tissue regeneration (Dhabhar 2014).

KEGG‐based functional profiling echoed these trends, though with reduced resolution compared to Reactome analysis. Carbon and pyruvate metabolism emerged as consistently enriched across all populations. These central metabolic pathways provide key energy intermediates and are often upregulated in response to xenobiotic exposure to fuel detoxification and repair mechanisms (Kshatriya and Gershenzon 2024). Their activation across contrasting population histories suggests a fundamental metabolic reprogramming in response to PHE, independent of prior environmental stress. Together, these results highlight the interplay between historical exposure and chemical stress responses, demonstrating how historical environmental stress shapes the extent and nature of transcriptomic plasticity to acute chemical stress. They also suggest that while some populations exhibit specialised responses evolved from prior exposures to pollution, core metabolic pathways are conserved across evolutionary and ecological contexts. These findings are consistent with those of Abdullahi, Zhou, et al. (2022), who demonstrated that long‐term chemical exposure erodes genetic diversity and diminishes stress responsiveness in *Daphnia* populations.

Our study is the first to examine the impact of hydrocarbon exposure on *Daphnia* gut microbiota diversity and function across populations with different environmental histories. Using two 16S rRNA regions (V1 and V4), we show that microbial diversity, composition, and predicted functions are shaped by both prior contaminant exposure and baseline community structure.

Our study extends the limited body of work on microbiome‐mediated responses to hydrocarbons in aquatic invertebrates (Ma et al. 2016) and aligns with findings in fish, where PAH exposure induces dysbiosis and enriches metabolic and degradation pathways (Bagi et al. 2018; González‐Penagos et al. 2020; Walter et al. 2019). These parallels suggest conserved microbial responses to PAH pollution across aquatic hosts. However, different taxonomic groups appear to employ distinct coping mechanisms: fish studies report reduced alpha diversity and dominance of hydrocarbon‐degrading taxa, whereas in *Daphnia*, increased detoxification capacity is accompanied by higher alpha diversity.

The microbiome of the semi‐pristine population exhibited upregulation of biosynthetic and structural pathways following PHE exposure, including amino acid and cofactor synthesis, peptidoglycan biosynthesis, and cell cycle regulation, indicating enhanced anabolic activity in response to acute stress. In contrast, downregulation of the ribosome pathway suggests reduced protein synthesis, likely reflecting microbial community restructuring. This combination of activated core metabolism and constrained growth is consistent with a microbiota not previously exposed to chemical stress but capable of mounting a generalised stress response.

The Eutrophic population displayed a complex functional profile, with both up‐ and downregulated pathways across metabolic and stress‐response domains. Notably, upregulation of toluene degradation and related metabolic pathways suggests enhanced microbial capacity for pollutant degradation, membrane integrity, and stress tolerance (Ren and Manefield 2025). In contrast, downregulation of porphyrin metabolism indicates a shift away from standard energy processing. These patterns are consistent with legacy adaptations to chronic eutrophication, reflecting a microbiome dominated by stress‐resilient and pollutant‐degrading taxa.

The Pesticides population exhibited the most extensive microbial functional response, with broad upregulation of energy‐generating and biosynthetic pathways, including carbon, butanoate, and pyruvate metabolism, as well as cofactor and amino acid biosynthesis. These shifts suggest increased metabolic demand to support microbial resilience and host detoxification. In contrast, glyoxylate and dicarboxylate metabolism was downregulated, indicating a shift away from energy‐conserving processes toward more energetically intensive functions, consistent with previous findings on pesticide‐driven microbiome adaptation in *Daphnia* (Janssens et al. 2022).

The recovery population showed a moderate but coordinated microbial response, marked by upregulation of carbohydrate‐related pathways including starch and sucrose metabolism, galactose metabolism, fructose and mannose metabolism, and the phosphotransferase system. These shifts suggest increased carbohydrate utilisation and efficient sugar transport, supporting microbial growth and energy balance during recovery from prior environmental stress. The absence of significantly downregulated pathways indicates a rebuilding microbiome focused on resource acquisition and structural maintenance (Deutscher et al. 2006).

## 5 Conclusions

This study demonstrates that sub‐lethal PHE exposure triggers coordinated, functional responses in both *D. magna* and its gut microbiota. Shared enrichment of central carbon and pyruvate metabolism across transcriptomic and microbiome datasets indicates a convergent shift toward energy mobilisation and detoxification under chemical stress. Enrichment of oxidative stress responses, xenobiotic metabolism pathways (e.g., cytochrome P450, glutathione), and biosynthesis of structural components, such as the cuticle in the host and membranes in the microbiota, reflects a systemic, multi‐layered defence strategy. While these patterns reveal tightly coordinated host–microbiome responses to chemical stress, they represent associative evidence; establishing causal relationships would require integrative, causal modelling approaches that link host co‐expression modules to microbial predicted functions using causative AI‐based inference frameworks (Suppa et al. 2020).

Population‐specific responses revealed that historical environmental exposure to anthropogenic pollutants shapes both host and microbial functional potential. It allowed us to distinguish between genetically encoded (constitutive) responses and plastic responses to acute toxicant exposure. This distinction is rarely captured in ecotoxicological studies and provides valuable insights into how evolutionary and environmental legacies shape both host and microbiome adaptability. The finding that population‐specific baselines modulate the magnitude and direction of plastic functional responses in both datasets suggests that long‐term exposure to environmental contaminants not only alters the host genome and physiology, as previously demonstrated (Abdullahi, Zhou, et al. 2022; Gigl et al. 2024; Rogalski 2015), but also rewires the structure and function of associated microbial communities.

By integrating transcriptomic and microbiome functional profiling, we provide a holistic understanding of how host‐microbe systems respond to environmental stress. This approach advances our ability to predict the evolutionary trajectories of chemical tolerance and highlights the gut microbiome as a key but underappreciated component in ecological risk assessments.

## Author Contributions

F.G.: Exposure experiments, first manuscript draft, microbiome data analysis, manuscript writing. M.A. and N.E.: Microbiome data analysis and visualisation, manuscript writing. S.B.B.: Transcriptome data analysis. J.Z.: Supervision, review and editing. H.H.: Funding, supervision, review and editing. L.O.: Conceptualisation, supervision, funding, coordination, manuscript writing, review and editing.

## Funding

FG received support from the RobustNature Excellence Cluster Initiative (SynergyFund) and the Goethe University Academy for Early Career Researchers (GRADE), which enabled him to complete the current study at the University of Birmingham. LO and MA receive funding from the Horizon Europe Innovation Actions under Grant Agreement No. 101112877 (UPSTREAM). LO and NE receive funding from the European Union under the Horizon Europe grant Partnership for the Assessment of Risk of Chemicals (PARC; 101057014). The results and conclusions reflect only the authors' view. The European Commission cannot be held responsible for any use that may be made of the information contained therein.

## Conflicts of Interest

The authors declare no conflicts of interest.

## Supporting information


**Table S1:** mec70272‐sup‐0001‐TablesS1‐S4.zip.

## Data Availability

The data that support the findings of this study are openly available in International Nucleotide Sequence Database Collaboration at https://www.ncbi.nlm.nih.gov/, reference number PRJNA1289021 an dPRJNA1289019.
