# Supplementary material for: Challenging Evolutionary Paradigms: Daphnia Populations Resurrected From Unpolluted Environments Show Enhanced Detoxification Ability to Aromatic Pollutants
Source: Mol Ecol. 2026 Feb 17;35(4):e70272. doi: 10.1111/mec.70272 (PMC12914093; doi:10.1111/mec.70272)

**Challenging evolutionary paradigms: *Daphnia* populations resurrected from unpolluted environments show enhanced detoxification ability to aromatic pollutants**

Florian Gigl^1,2^, Muhammad Abdullahi^2^, Sam Benkwitz-Bedford^2^, Niamh Eastwood^2^, Jiarui Zhou^2^, Henner Hollert^1,3,4*^, Luisa Orsini^1,2,5*^

^1^Department of Evolutionary Ecology and Environmental Toxicology, Faculty of Biological Sciences, Goethe University, Max-von-Laue-Straße 13, 60438 Frankfurt am Main, Germany

^2^ School of Biosciences and Centre for Environmental Research and Justice (CERJ), University of Birmingham. Birmingham, B15 2TT UK

^3^Department Environmental Media Related Ecotoxicology, Fraunhofer Institute for Molecular Biology and Applied Ecology IME, Auf dem Aberg 1, 57392 Schmallenberg, Germany

^4^LOEWE Centre for Translational Biodiversity Genomics (LOEWE-TBG), Senckenberganlage 25, 60325 Frankfurt am Main

^5^The Alan Turing Institute, British Library, 96 Euston Road. London NW1 2DB

*these authors share senior authorship

Corresponding Author:

Luisa Orsini (l.orsini@bham.ac.uk)

**Supplementary tables and figures**

**Table S1. Gene read counts.** Table showing the raw read counts for each gene across individual *Daphnia* *magna* genotypes used in this study. Counts represent the number of sequencing reads mapped to each gene (GeneID) following quality control and mapping onto the reference genome.

*See Gigl et al. Table S1*

**Table S2. Pathway analysis.** This multitab file includes pathways identified in the overrepresentation analysis using the Reactome database for *Daphnia* populations (Tab 1) and genotypes (Tab 2), as well as enriched pathways identified in the KEGG database for the *Daphnia* populations (Tab 3) and genotypes (Tab 4). All Tabs include Pathway name, presence/absence in the *Daphnia* populations/genotype, functional classification and functional process for each identified pathway.

*See Gigl et al. Table S2*

**Table S3.** ***Post hoc* PERMANOVA results** comparing gut microbial community composition across treatment groups and *Daphnia* populations. Pairwise comparisons include (i) treated vs. control within each population and (ii) between populations, for both control (CO) and phenanthrene-treated (PHE) groups. Results are based on Bray–Curtis dissimilarity and 999 permutations. Significant differences (p < 0.05) are in bold.

| Treatment | | | | | | | |
| --- | --- | --- | --- | --- | --- | --- | --- |
| 16S V1 | | | | 16S V4 | | | |
| Group1 | Group2 | R2 | *P-val* | Group1 | Group2 | R2 | *P-val* |
| Eutrophic-CO | Eutrophic-PHE | 0.0988 | 0.129 | Eutrophic-CO | Eutrophic-PHE | 0.054783 | 0.542667 |
| Recovery-CO | Recovery-PHE | 0.19872 | **0.04133** | Recovery-CO | Recovery-PHE | 0.160607 | 0.426 |
| Semi-Pristine-CO | Semi-Pristine-PHE | 0.14084 | **0.016** | Semi-Pristine-CO | Semi-Pristine-PHE | 0.026761 | 0.566 |
| Pesticide-CO | Pesticide-PHE | 0.24883 | **0.026** | Pesticide-CO | Pesticide-PHE | 0.328104 | **0.056** |
| Population | | | | | | | |
| 16S V1 | | | | 16S V4 | | | |
| Group1 | Group2 | R2 | *P-val* | Group1 | Group2 | R2 | *P-val* |
| Eutrophic | Recovery | 0.05261 | 0.258 | Eutrophic | Recovery | 0.03484 | 0.432 |
| Eutrophic | Semi-Pristine | 0.03791 | 0.237 | Eutrophic | Semi-Pristine | 0.02134 | 0.432 |
| Eutrophic | Pesticide | 0.08988 | **0.054** | Eutrophic | Pesticide | 0.05003 | 0.432 |
| Recovery | Semi-Pristine | 0.06616 | 0.068 | Recovery | Semi-Pristine | 0.03594 | 0.432 |
| Recovery | Pesticide | 0.05109 | 0.364 | Recovery | Pesticide | 0.05783 | 0.432 |
| Semi-Pristine | Pesticide | 0.08766 | **0.042** | Semi-Pristine | Pesticide | 0.05539 | 0.432 |

**Table S4. Enriched KEGG pathways from the microbial functional profiles.** Pathways identified through over-representation analysis based on differentially abundant KEGG Orthologs (KOs) predicted using PICRUSt2. Pathways are classified hierarchically by functional domain, functional subsystem, and specific pathway function, along with the corresponding KEGG Pathway ID. The number of genes contributing to each pathway, associated KO identifiers, unadjusted *P*-values, and FDR-adjusted *P*-values (P *adj*) are included. Direction indicates whether the pathway was up- or downregulated in PHE-exposed samples relative to their controls. The associated *Daphnia* population and 16S marker gene region (V1 or V4) used for inference are also noted.

*See Gigl et al. Table S4*

**Figure S1. PCA plots**. Principal Component Analysis (PCA) plots showing the variance explained by the first two components. Overall, the first three components explain up to 52% of the total variance. Each point represents one of 11 Daphnia genotypes (with three biological replicates each), grouped by population: Semi-pristine (black), Eutrophic (blue), Pesticides (green), and Recovery (red).


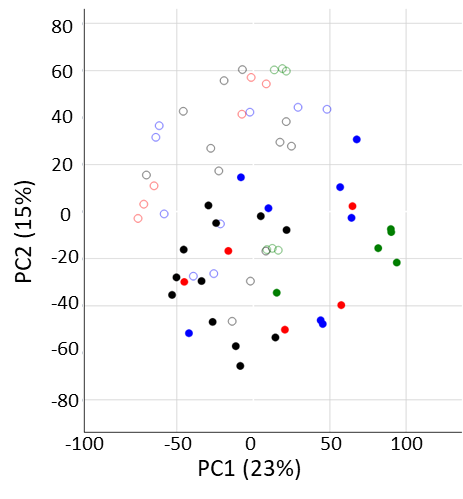


**Figure S2. Pathways enriched in the *Daphnia* genotypes.** Total number of pathways identified in the *Daphnia* genotypes with the (A) overrepresentation analysis (Reactome) and (B) the enrichment analysis (KEGG). The black colour indicates the presence, whereas the white colour indicates the absence of a certain pathway in any given *Daphnia* genotype. The complete list of identified pathways across the *Daphnia* genotypes in the two databases is in Table S2.


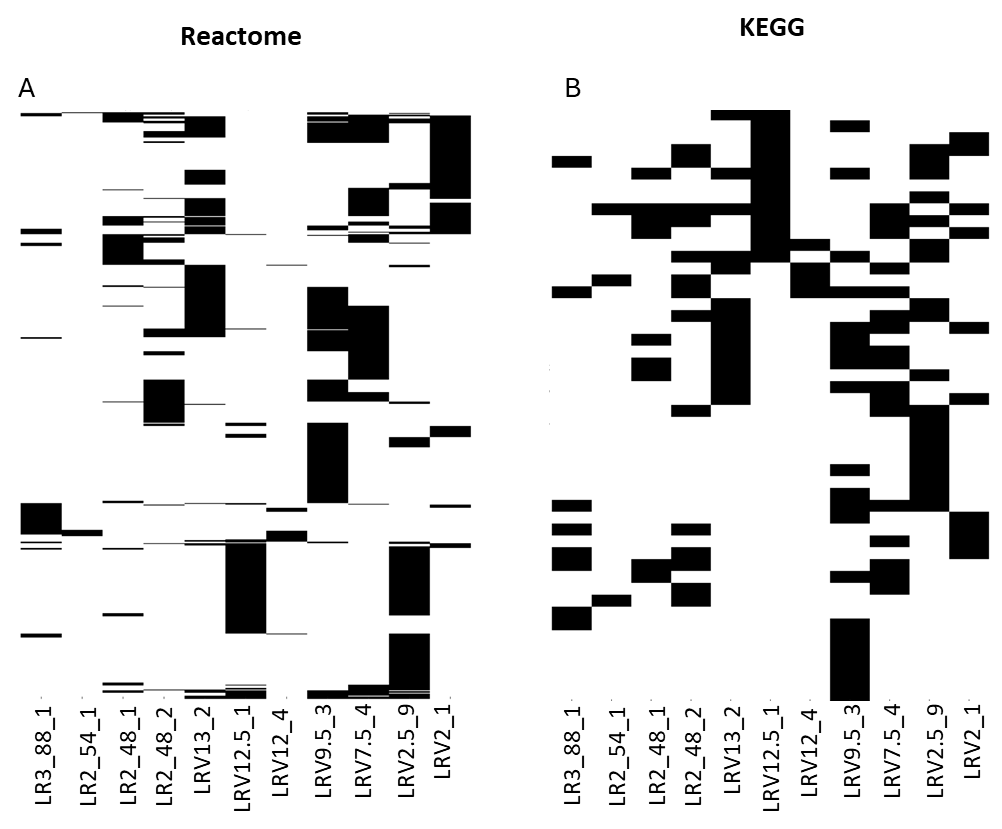


**Figure S3. Taxa barplots for genera.** Topmost abundant genera in the *Daphnia* populations gut microbiome for the 16SV1 (A) and 16SV4 (B) gene marker.


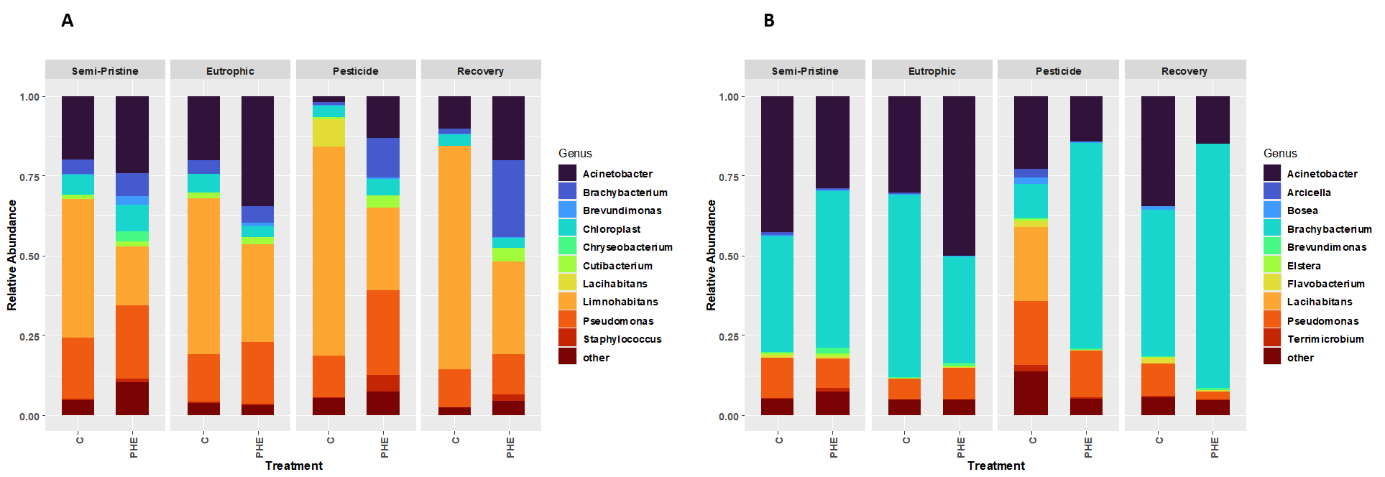

Supplement: Supplementary file 1 — Table S1: mec70272‐sup‐0001‐TablesS1‐S4.zip. [file MEC-35-e70272-s001.zip › mec70272-sup-0001-TableS1-S4-FigureS1-S3@Gigl_etal_Supporting information R2.docx]
